# A Cas9 with PAM recognition for adenine dinucleotides

**DOI:** 10.1038/s41467-020-16117-8

**Published:** 2020-05-18

**Authors:** Pranam Chatterjee, Jooyoung Lee, Lisa Nip, Sabrina R. T. Koseki, Emma Tysinger, Erik J. Sontheimer, Joseph M. Jacobson, Noah Jakimo

**Affiliations:** 1 Center for Bits and Atoms, Cambridge, MA United States; 2 Media Lab, Massachusetts Institute of Technology, Cambridge, MA United States; 3 RNA Therapeutics Institute, University of Massachusetts Medical School, Worcester, MA United States

**Keywords:** Synthetic biology, Computational biology and bioinformatics, CRISPR-Cas9 genome editing

## Abstract

CRISPR-associated (Cas) DNA-endonucleases are remarkably effective tools for genome engineering, but have limited target ranges due to their protospacer adjacent motif (PAM) requirements. We demonstrate a critical expansion of the targetable sequence space for a type II-A CRISPR-associated enzyme through identification of the natural 5$^{\prime}$-NAAN-3$^{\prime}$ PAM preference of *Streptococcus macacae* Cas9 (SmacCas9). To achieve efficient editing activity, we graft the PAM-interacting domain of SmacCas9 to its well-established ortholog from *Streptococcus pyogenes* (SpyCas9), and further engineer an increased efficiency variant (iSpyMac) for robust genome editing activity. We establish that our hybrids can target all adenine dinucleotide PAM sequences and possess robust and accurate editing capabilities in human cells.

## Abstract (web summary)

Protospacer adjacent motif (PAM) requirements limit the target range of CRISPR endonucleases. Here, the authors graft the 5$^{\prime}$-NAAN-3$^{\prime}$ PAM-interacting domain of SmacCas9 onto SpyCas9 to create adenine dinucleotide targeting chimeras.

## Introduction

Biotechnologies based on RNA-guided CRISPR systems have enabled precise and programmable genomic interfacing^1^. However, CRISPR-associated (Cas) endonucleases are also collectively restrained from localizing to any position along double-stranded DNA (dsDNA) due to their requirement for targets to neighbor a protospacer adajacent motif (PAM)^2–4^. Current gaps in the PAM sequences that Cas enzymes are known to recognize prevent access to numerous genomic positions for powerful genome editing activities, such as base editing, prime editing, and homology-directed repair^5–8^. Many adenine-thymine (AT)-rich regions, in particular, have been excluded from compelling CRISPR applications because previously reported endonucleases, such as Cas9 and Cas12a (formerly known as Cpf1), require targets to neighbor guanine-cytosine (GC)-content or more restrictive motifs, respectively^9–11^.

In this work, we introduce an ortholog of the well-established Cas9 from *Streptococcus pyogenes* (SpyCas9), derived from *Streptococcus macacae NCTC 11558*, that can instead recognize a short 5$^{\prime}$-NAA-3$^{\prime}$ PAM^12^. These sequences constitute 18.6% of the human genome, making adjacent adenines the most abundant dinucleotide (Supplementary Fig. 1A–B). The importance of this alternative PAM recognition for a Cas9 enzyme is reinforced by recent work exposing that Type-V DNA-targeting CRISPR nucleases (including Cas12 and Cas14 orthologs), while targeting dsDNA at AT-rich PAM sites with intrinsic high fidelity, will indiscriminately digest single-stranded DNA (ssDNA) once bound to their targets^13–16^. Such collateral activity may introduce unwanted risks around partially unpaired chromosomal structures, such as transcription bubbles, R-loops, and replication forks.

Here we present engineered nucleases derived from SmacCas9 and characterize their altered specificity and utility by means of transcriptional repression in bacterial culture, in vitro digestion reactions, and gene editing activity in human cells. Our results demonstrate complete 5$^{\prime}$-NAAN-3$^{\prime}$ PAM recognition of our engineered variants in all tested contexts.

## Results

### Discovery of SmacCas9

**Fig. 1:**
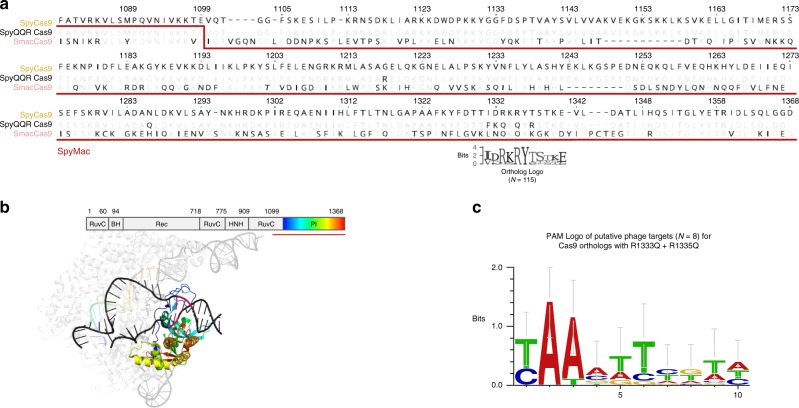
Identification of features from natural PAM divergence through bioinformatics. **a** Sequence alignment of SpyCas9, its QQR variant, and SmacCas9. The step in the underlining red line marks the joining of SpyCas9 and SmacCas9 to construct a SpyMac hybrid. The sequence logo (Weblogo online tool) immediately below the alignment depicts the conservation at 11 positions around the PAM-contacting arginines of SpyCas9. **b** The domain organization of SpyCas9 juxtaposed over a color-coded structure of RNA-guided, target-bound SpyCas9 (PDB ID 5F9R). The two DNA strands are black with the exception of a magenta segment corresponding to the PAM. A blue–green–red color map is used for labeling the Cas9 PI domain and guide spacer sequence to highlight structures that confer sequence specificity and the prevalence of intra-domain contacts within the PI^43^. **c** A sequence logo generated online (WebLogo) that was input with putative PAM sequences found in *Streptococcus phage* and associated with close SmacCas9 homologs.

To modify the ancestral 5$^{\prime}$-NGG-3$^{\prime}$ PAM specificity of SpyCas9, early and recent reports have employed directed evolution (e.g., "VQR”, "EQR”, "VRER”, and "NRNH” variants) or rational design informed by crystal structure (e.g., "QQR”, "NG”, and "NR” variants)^17–22^. These reports focused on the PAM-contacting arginine residues R1333 and R1335 that abolish function when exclusively mutated. While those studies identified compensatory mutations resulting in altered PAM specificity, the Cas9 variants that they produced maintained a guanine preference in at least one position of the PAM sequence for reported in vivo editing. Concurrent reports have used evolutionary information to further relax the canonical 5$^{\prime}$-NNGRRT-3$^{\prime}$ PAM specificity of *Staphylococcus aureus* Cas9 (SaCas9) or to discover alternative 5$^{\prime}$-NNNNCC-3$^{\prime}$ PAM specificity to the canonical 5$^{\prime}$-NNNNGHTT-3$^{\prime}$ PAM of *Neisseria meningitidis* Cas9^23,24^. The nucleases from both of these new reports, however, still prefer GC-content in at least one position of the PAM sequence. We aimed to lift such GC-content prerequisites via a custom bioinformatics-driven workflow that mines existing PAM diversity in the *Streptococcus* genus^25^. Using this strategy, we homed in on SmacCas9 as having the potential to bear altered non-GC PAM specificity upon aligning 115 orthologs of SpyCas9 from UniProt (limited to those with greater than a 70% pairwise BLOSSOM62 score). From the alignment we found SmacCas9 was one of two close homologs, along with a *Streptococcus mutans B112SM-A* Cas9 (SmutCas9), possessing glutamines at both of the positions aligned to the otherwise highly conserved PAM-contacting arginines (Fig. 1a–b; Supplementary Fig. 2A). Arginine residues are known to strongly prefer guanines in the amino-acid-base interaction landscape, as evidenced by the 5$^{\prime}$-NGG-3$^{\prime}$ specificity of SpyCas9. Glutamine residues, on the other hand, preferentially bind to adenines, through interaction with the major groove edge^26^. We thus hypothesized that SmacCas9 had naturally coevolved the necessary compensatory mutations to gain new adenine-rich PAM recognition. A small sample size of 13 spacers from its corresponding genome’s CRISPR array prevented us from confidently inferring the SmacCas9 PAM in silico. Nevertheless, the possibility for SmacCas9 requiring less GC-content in its PAM was supported by sequence similarities to the "QQR” variant that has 5$^{\prime}$-NAAG-3$^{\prime}$ specificity^27^, in addition to the AT-rich putative consensus PAM for phage-originating spacers in CRISPR arrays associated with highly homologous SmutCas9, which were identified with the aid of our previously-described SPAMALOT pipeline and consistent with previous predictions (Fig. 1c; Supplementary Fig. 2B; Supplementary Fig. 3) ^25,28^.

### Engineering and PAM characterization of SpyMac

**Fig. 2:**
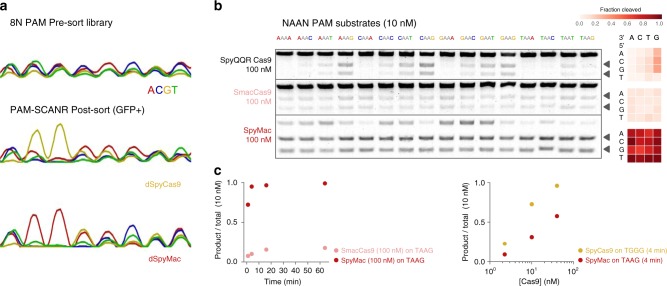
Validation of SmacCas9 recognition for adenine dinucleotide PAM sequences. **a** Chromatograms representing the PAM-SCANR based enrichment of variant-recognizing PAM sequences from a 5$^{\prime}$-NNNNNNNN-3$^{\prime}$ library. **b** SYBR-stained agarose gels showing in vitro digestion of 10 nM 5$^{\prime}$-NAAN-3$^{\prime}$ substrates upon 16 minutes of incubation with 100 nM of purified ribonucleoprotein enzyme assemblies. Arrows distinguish banding of the cleaved products from uncleaved substrate (top band). Matrix plots summarize cleaved fraction calculations, which were carried out in a custom script for processing gel images. Samples were performed in independent biological duplicates (*n* = 2). **c** Time course measurements of target DNA substrate cleavage for SmacCas9 and SpyMac. **d** DNA substrate cleavage plotted as a function of 0.25:1, 1:1, and 4:1 molar ratios of ribonucleoprotein to target for wild-type SpyCas9 and hybrid SpyMac. Source data are provided as a Source Data file.

We proceeded to empirically determine the PAM preferences of several *Streptococcus* orthologs that change one or both of the critical PAM-contacts. Based on demonstrated examples of the PAM-interaction domain (PID) and guide RNA (gRNA) having cross-compatibility between Cas9 orthologs that are closely related and active, we constructed new variants by rationally exchanging the PI region of catalytically "dead” SpyCas9 (dSpyCas9) with those of the selected orthologs (Supplementary Fig. 2A–B)^29,30^. Assembled variants, including dSpyMacCas9 (herein referred to as dSpyMac), were separately cotransformed into *E. coli* cells, along with guide RNA derived from *S. pyogenes* and an 8-mer PAM library of uniform base representation in the PAM-SCANR genetic circuit, established by Leenay et al.^31^. The circuit upregulates a green fluorescent protein (GFP) reporter in proportion to PAM-binding strength. Therefore, we collected the GFP-positive cell populations by flow cytometry (Supplementary Fig. 4) and Sanger sequenced them around the site of the PAM to determine position-wise base preferences in a corresponding variant’s PAM recognition. dSpyMac, more so than dSpyMutCas9, generated a trace profile that was most consistent with guanine-independent PAM recognition, along with a dominant specificity for adenine dinucleotides (Fig. 2a; Supplementary Fig. 2C).

Next, we purified nuclease-active enzymes to continue probing the DNA target recognition potential and uniqueness of SpyMac. (Supplementary Fig. 5A)^27,32^. We individually incubated the ribonucleoprotein complex enzymes (composed of Cas9 + crRNA + tracrRNA) with double-stranded target substrates of all 5$^{\prime}$/3$^{\prime}$-neighboring base combinations at an adenine dinucleotide PAM (5$^{\prime}$-NAAN-3$^{\prime}$; Fig. 2b). A brief 16-min digestion indicated both wild-type SmacCas9 and the hybrid SpyMac cleaved adjacent to 5$^{\prime}$-NAAN-3$^{\prime}$ motifs more broadly and evenly than the previously reported QQR variant. SpyMac distinguished itself further with rapid DNA-cutting rates that resemble the fast digest kinetics of SpyCas9 (Fig. 2c–d)^33^. We ran reactions that used varying crRNA spacer lengths and tracrRNA sequence, as the latter differs slightly between the *S. macacae* and *S. pyogenes* genomes (Supplementary Fig. 5B–E). Neither of these two parameters compensated for the slower cleavage rate of SmacCas9, but we did notice marginal improvement in the activity of the wild-type form with its native tracrRNA, which comports with the interface of the guide-Cas9 interaction being mostly outside of the PI domain.

To verify that an adenine dsDNA dinucleotide is sufficient for Cas9 PAM recognition and target cleavage, we assembled target sequences that switch the next four downstream bases to the same nucleotide (e.g., 5$^{\prime}$-TAAGXXXX-3$^{\prime}$, for X all fixed to A, C, G, or T; Supplementary Fig. 5F). We confirmed SpyMac remains active across this target set, albeit with some variation in cutting rate. Additionally, we observed moderate yield of cleaved products on examples of 5$^{\prime}$-NBBAA-3$^{\prime}$, 5$^{\prime}$-NABAB-3$^{\prime}$, 5$^{\prime}$-NBABA-3$^{\prime}$ PAM sequences (where B is C, G, or T; Supplementary Fig. 5G), revealing an even broader tolerance for increments to the dinucleotide position or adenine adjacency. We anticipate future measurements of guide-loading, target-dissociation and R-loop expansion/contraction will provide more insights on the serendipitous catalytic benefit over SmacCas9 from grafting its PI domain onto a truncated SpyCas9.

### Genome editing with iSpyMac

**Fig. 3:**
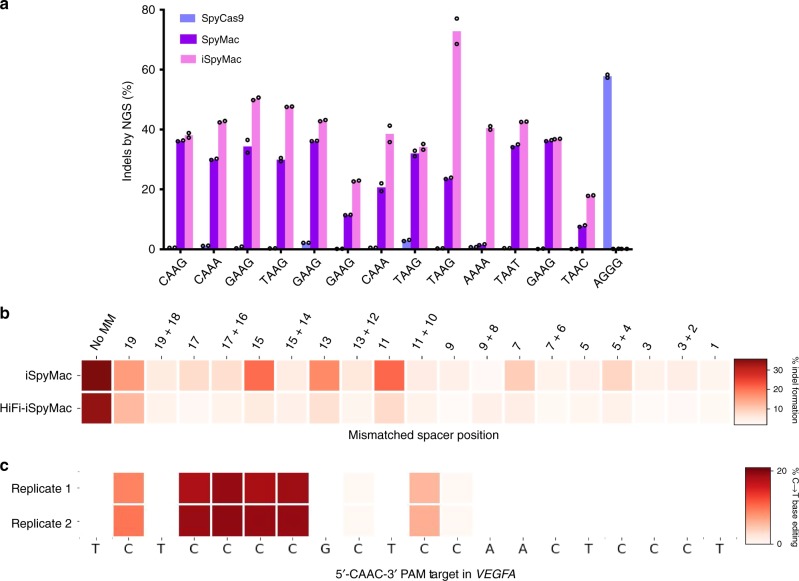
Genome editing capabilities of engineered SmacCas9 variants. **a** CRISPResso2 indel analysis following NGS of amplified genomic regions to assess on-target editing of iSpyMac in comparison to SpyMac and SpyCas9, on the indicated 5$^{\prime}$-NAA-3$^{\prime}$ and 5$^{\prime}$-NGG-3$^{\prime}$ PAM sequences. Samples were performed in two independent transfection replicates (*n* = 2). **b** Efficiency heatmap of mismatch tolerance assay on genomic targets. Quantified indel frequencies, as assessed by the TIDE algorithm^41^, are exhibited for each labeled single or double mismatch in the sgRNA sequence for the indicated Cas9 variant and indicated PAM sequence. Samples were performed in two independent transfection replicates (*n* = 2). **c** CRISPResso2 genomic base editing analysis following NGS of genomic amplicons to assess conversion of cytosines to thymines by iSpyMac-BE3. Samples were performed in two independent transfection replicates (*n* = 2). All source data are provided as a Source Data file.

To assess the altered 5$^{\prime}$-NAAN-3$^{\prime}$ targeting capabilities of SpyMac, as opposed to SpyCas9, we cotransfected HEK293T cells with plasmids expressing these nucleases alongside one of 12 sgRNAs, targeting PAMs with varying combinations of bases flanking the adenine dinucleotide, within 5 distinct genomic loci. After 5 days post-transfection, we extracted genomic DNA, amplified the target loci, and quantified indel frequencies via next-generation sequencing (NGS). Our results first demonstrate that while SpyCas9 is able to achieve over a 50% modification rate on the 5$^{\prime}$-NGGN-3$^{\prime}$ substrate, it has negligible activity on 5$^{\prime}$-NAAN-3$^{\prime}$ targets. Alternatively, SpyMac achieves modification on most, but not all, tested 5$^{\prime}$-NAAN-3$^{\prime}$ loci. In fact, SpyMac demonstrated negligible editing on the tested 5$^{\prime}$-AAAA-3$^{\prime}$ target sequence within the *PVALB* gene. To address sites with low modification efficiencies, we introduced two mutations (R221K and N394K) into SpyMac that had been previously identified by deep mutational scans of SpyCas9 to increase editing efficiency^34^. We refer to this variant as an increased editing SpyMac (iSpyMac) due to its elevated modification efficiencies on all tested 5$^{\prime}$-NAAN-3$^{\prime}$ targets, even editing on sites that SpyMac is unable to access, such as 5$^{\prime}$-AAAA-3$^{\prime}$ PAM sites (Fig. 3a). Additionally, iSpyMac exhibits comparable modification efficiencies to other A-rich CRISPR nucleases, such as Cas12a effectors from *Acidaminococcus sp*. and *Lachnospiraceae bacterium ND2006* (Supplementary Fig. 6).

Next, we assessed the tolerance of iSpyMac to mismatched sequences, by employing sgRNAs harboring double or single mismatches to a fixed protospacer within the *AAVS* gene, possessing a 5$^{\prime}$-GAAG-3$^{\prime}$ PAM sequence. iSpyMac demonstrated editing capabilities on targets with single mismatches within the PAM-proximal segment of the sgRNA. To mitigate this supposed off-target propensity, we introduced the R691A mutation, which was previously isolated via bacterial selection for SpyCas9 to maintain high on-target activity while reducing off-target editing^35^. Our high-fidelity variant, HiFi-iSpyMac, exhibited nearly negligible activity on mismatched sequences, as compared to the original enzyme, with minimal loss of on-target activity (Fig. 3b).

Lastly, we selected a window of four nucleotides in the *VEGFA* locus in a sequence context such that any other CRISPR endonuclease with reported use for base editing would not allow their base editing with a cytidine deaminase-fused enzyme^36^. Accordingly, we cotransfected HEK293T cells with a nickase form of iSpyMac derived from the previously reported BE3 architecture for cytosine base editing (iSpyMac-BE3) and the sgRNA plasmid targeting a PAM downstream of the selected nucleotides^5^. We measured effective base editing levels in harvested cells, which exhibited over 20% cytosine to thymine conversion at these positions via NGS analysis (Fig. 3c).

## Discussion

In summary, we have identified a homolog of SpyCas9 in *Streptococcus macacae* with native 5$^{\prime}$-NAAN-3$^{\prime}$ PAM specificity. By leveraging the high similarity in Cas9 sequences between different *Streptococcus* species and the substantial background in the development and characterization of SpyCas9, we have engineered variants of SmacCas9 that maintain its minimal adenine dinucleotide PAM specificity and achieve efficient and accurate activity for mediating edits on chromosomes in human cells^37^. This finding sets the path for engineering enzymes like iSpyMac with other desirable properties, control points, effectors, and activities^8,38–40^. iSpyMac can now open wide access to AT-content PAM sequences in the ever-growing list of genome engineering applications with type II-A CRISPR-Cas systems.

## Methods

### Selection of *Streptococcus* Cas9 orthologs of interest

All Cas9 orthologs from the *Streptococcus* genus were downloaded from the online UniProt database https://www.uniprot.org/. These were the downselected by pairwise alignment to SpyCas9 using a BLOSUM62 cost matrix in Genewiz software, discarding orthologs with less than 70% agreement with the Spy Cas9 sequence. The remaining 115 orthologs were used to generate a sequence logo (Weblogo http://weblogo.threeplusone.com/create.cgi), and were manually selected for divergence at positions aligned to residues critical for the PAM interaction of SpyCas9. The SPAMALOT pipeline was implemented as we previously reported^25^. Briefly, a set of scripts based around the Bowtie alignment tool (http://bowtie-bio.sourceforge.net) map the spacer sequences from CRISPR cassettes to putative targets in phage genomes. The SPAMALOT software can be downloaded at https://github.com/mitmedialab/SPAMALOT.

### PAM-SCANR bacterial fluorescence assay

Sequences encoding the PAM-interaction domains of selected Cas9 orthologs were synthesized as gBlock fragments by Integrated DNA Technologies (IDT) and inserted via a New England Biolabs (NEB) Gibson Assembly reaction into the C-terminus of a low-copy plasmid containing dSpyCas9 (Beisel Lab, NCSU). The hybrid-protein constructs were transformed into electrocompetent *E. coli* cells with additional PAM-SCANR components as previously established^31^. Overnight cultures were analyzed and sorted on a Becton Dickinson (BD) FACSAria machine. Sorted GFP-positive cells were grown to sufficient density, and plasmids from the pre-sorted and sorted populations were then isolated. The region flanking the nucleotide library was PCR-amplified and submitted for Sanger sequencing (Genewiz). The chromatograms from received trace files were inspected for post-sorted sequence enrichments relative to the pre-sorted library.

### Purification of and DNA cleavage with selected nucleases

The gBlock (IDT) encoding the PAM-interaction domain of *S. macacae* was inserted into a bacterial protein expression/purification vector containing wild-type *S. pyogenes* Cas9 fused to the His6-MBP-tobacco etch virus (TEV) protease cleavage site at the N-terminus (pMJ915 was a gift from Jennifer Doudna, Addgene plasmid #69090). The resulting hybrid SpyMac Cas9 protein expression construct was sequence-verified by a next-generation complete plasmid sequencing service (CCBI DNA Core Facility at Massachusetts General Hospital). The hybrid-protein construct was then transformed into BL21 Rosetta 2^TM^(DE3) (MilliporeSigma), and a single colony was picked for protein expression, inoculated in 1 L 2xYT media, and grown at 37 °C to a cell density of OD600 0.6. The temperature was then lowered to 18 °C and His-MBP-TEV-SpyMac Cas9 expression was induced by supplementing with 0.2 mM IPTG for an additional 18 h of growth before harvest. Cells were then lysed with BugBuster^TM^Protein Extraction Reagent, supplemented with 1 mg/ml lysozyme solution (MilliporeSigma), 125 Units/gram cell paste of Benzonase^TM^Nuclease (MilliporeSigma), and complete, EDTA-free protease inhibitors (Roche Diagnostics Corporation). The lysate was clarified by centrifugation, including a final spin with a prechilled Steriflip^TM^0.45 micron filter (MilliporeSigma). The clarified lysate was incubated with Ni-NTA resin (Qiagen) at 4 °C for 1 h and subsequently applied to an Econo-Pac^TM^chromatography column (Bio-Rad Laboratories). The protein-bound resin was washed extensively with wash buffer (20 mM Tris pH 8.0, 800 mM KCl, 20 mM imidazole, 10% glycerol, 1 mM TCEP) and His-tagged SpyMac protein was eluted in wash buffer (20 mM HEPES, pH 8.0, 500 mM KCl, 250 mM imidazole, 10% glycerol). ProTEV^TM^Plus protease (Promega, Madison) was added to the pooled fractions and dialyzed overnight into storage buffer (20 mM HEPES, pH 7.5, 500 mM KCl, 20% glycerol) at 4 °C using Slide-A-Lyzer^TM^dialysis cassettes with a molecular weight cutoff of 20 KDa (ThermoFisher Scientific). The sample was then incubated again with Ni-NTA resin for 1 h at 4 °C with gentle rotation and applied to a chromatography column to remove the cleaved His tag. The protein was eluted with wash buffer (20 mM Tris pH 8.0, 800 mM KCl, 20 mM imidazole, 10% glycerol, 1 mM TCEP) and fractions containing cleaved protein were verified once more by SDS-PAGE and Coomassie staining, then pooled, buffer exchanged into storage buffer, and concentrated. The concentrated aliquots were measured based on their light-absorption (Implen Nanophotometer) and flash-frozen at −80 °C for storage or used directly for in vitro cleavage assays. The crRNA and tracrRNA guide components were procured in the form of HPLC-purified RNA oligos (IDT) and resuspended in 1X IDTE pH 7.5 solution (IDT). Duplex crRNA-tracrRNA guides were annealed at 1 uM concentration in duplex buffer (IDT) by a protocol of rapid melting followed by gradual cooling. Target substrates were PCR-amplified from assemblies of the PAM-SCANR plasmid with a fixed PAM sequence. In vitro digestion reactions with 10 nM target and typically a 10-fold excess of enzyme components were prepared on ice and then incubated in a thermal cycler at 37 °C. Reactions were halted after at least 1 min of incubation by subsequent heat denaturation at 65 °C for 5 min and run on a 2% TAE-agarose gel stained with DNA-intercalating SYBR dye (Invitrogen). Gel images were recorded from blue-light exposure and analyzed in a Python script adapted from https://github.com/jharman25/gelquant/. Cleavage fraction measurements were quantified, in ImageJ (imagej.nih.gov) by the relative intensity of substrate and product bands as follows:

$$\% \,{\rm{cleaved}}\,{\rm{fraction}}\;=\;\frac{{\rm{Integrated}}\,{\rm{intensity}}\,{\rm{of}}\,{\rm{product}}\,{\rm{bands}}}{{\rm{Integrated}}\,{\rm{intensity}}\,{\rm{of}}\,{\rm{all}}\,{\rm{bands}}}$$

### Cell culture and DNA modification analysis

HEK293T cells were maintained in DMEM supplemented with 100 units/ml penicillin, 100 mg/ml streptomycin, and 10% fetal bovine serum (FBS). sgRNA plasmids (100  ng) and nuclease plasmids (100 ng) were transfected into cells as duplicates (2 × 10^4^/well in a 96-well plate) with Lipofectamine 3000 (Invitrogen) in Opti-MEM (Gibco). After 5 days post-transfection, genomic DNA was extracted using QuickExtract Solution (Epicentre), and genomic loci were amplified by PCR utilizing the Phusion Hot Start Flex DNA Polymerase (NEB). Amplicons were enzymatically purified and submitted for Sanger sequencing or NGS sequencing. Sanger sequencing ab1 files were analyzed using the TIDE algorithm (tide.deskgen.com)^41^. in comparison to an unedited control to calculate indel frequencies. NGS FASTQ files were analysed using a batch version of the software CRISPResso2 (https://github.com/pinellolab/CRISPResso2)^42^. All samples were performed in independent duplicates (*n* = 2). Standard deviation was used to calculate error bars.

### Statistical analysis

Data are shown as the mean of duplicate values, which are indicated by dots for each figure. Data were plotted using Matplotlib and the GraphPad Prism software (graphpad.com/scientific-software/prism/).

### Reporting summary

Further information on research design is available in the Nature Research Reporting Summary linked to this article.

## Supplementary information

Supplementary Information

Peer Review File

Reporting Summary

## Data Availability

Sequence data that support the findings of this study are available via the NIH Sequence Read Archive via BioProject PRJNA623926. Data underlying Figs. 2-3 and Supplementary Fig. 6 are provided as Source Data.

## Source data

Source Data
