# Supplementary material for: A Cas9 with PAM recognition for adenine dinucleotides
Source: Nat Commun. 2020 May 18;11:2474. doi: 10.1038/s41467-020-16117-8 (PMC7235249; doi:10.1038/s41467-020-16117-8)
Supplement: Supplementary file 3 — Reporting Summary [file 41467_2020_16117_MOESM3_ESM.pdf]

nature research

Corresponding author(s): Pranam Chatterjee

Last updated by author(s): Apr 8, 2020

Reporting Summary

Nature Research wishes to improve the reproducibility of the work that we publish. This form provides structure for consistency and transparency in reporting. For further information on Nature Research policies, see Authors & Referees and the Editorial Policy Checklist .

Statistics

For all statistical analyses, confirm that the following items are present in the figure legend, table legend, main text, or Methods section.

n/a	Confirmed	
☐	☒	The exact sample size (n) for each experimental group/condition, given as a discrete number and unit of measurement
☐	☒	A statement on whether measurements were taken from distinct samples or whether the same sample was measured repeatedly
☐	☒	The statistical test(s) used AND whether they are one- or two-sided<br>*Only common tests should be described solely by name; describe more complex techniques in the Methods section.*
☒	☐	A description of all covariates tested
☒	☐	A description of any assumptions or corrections, such as tests of normality and adjustment for multiple comparisons
☐	☒	A full description of the statistical parameters including central tendency (e.g. means) or other basic estimates (e.g. regression coefficient) AND variation (e.g. standard deviation) or associated estimates of uncertainty (e.g. confidence intervals)
☒	☐	For null hypothesis testing, the test statistic (e.g. F, t, r) with confidence intervals, effect sizes, degrees of freedom and P value noted<br>*Give P values as exact values whenever suitable.*
☒	☐	For Bayesian analysis, information on the choice of priors and Markov chain Monte Carlo settings
☒	☐	For hierarchical and complex designs, identification of the appropriate level for tests and full reporting of outcomes
☒	☐	Estimates of effect sizes (e.g. Cohen's d, Pearson's r), indicating how they were calculated

Our web collection on statistics for biologists contains articles on many of the points above.

Software and code

Policy information about availability of computer code

Data collection	Sanger sequencing data was collected as ab1 files from Genewiz. FASTQ files were collected from Genewiz for NGS data.
Data analysis	The TIDE algorithm ((v1) were used for indel analysis from Sanger Sequencing ab1 files. CRISPResso2 was used for NGS indel analysis. GraphPad Prism (v8) and Matplotlib (v3.2.1)was used to plot graphs. Gel images were analyzed by ImageJ (Version 1.52s). The Bowtie alignment tool (v1.2.3) and the SPAMALOT tool (v1) (https://github.com/mitmedialab/SPAMALOT) were used for sequence analysis.

For manuscripts utilizing custom algorithms or software that are central to the research but not yet described in published literature, software must be made available to editors/reviewers. We strongly encourage code deposition in a community repository (e.g. GitHub). See the Nature Research guidelines for submitting code & software for further information.

Data

Policy information about availability of data

All manuscripts must include a data availability statement. This statement should provide the following information, where applicable:

- Accession codes, unique identifiers, or web links for publicly available datasets
- A list of figures that have associated raw data
- A description of any restrictions on data availability

Sequence data that support the findings of this study are available via the NIH Sequence Read Archive via BioProject PRJNA623926. Data underlying Figs. 2-3 and Supplementary Fig. 6 are provided as Source Data.

Field-specific reporting

Please select the one below that is the best fit for your research. If you are not sure, read the appropriate sections before making your selection.

Life sciences study design

All studies must disclose on these points even when the disclosure is negative.

Sample size	Sample size was determine as n=2 to ensure consistent reproducibility across experiments.
Data exclusions	No data was excluded from the study. All values shown represent all data used in the study.
Replication	All samples were performed in independent biological duplicates (n=2) and all attempts at replication were successful.
Randomization	sgRNA sequences and PAMs were chosen randomly without any sequence bias, given that each contained the target AA at the correct position.
Blinding	Blinding is not relevant to this study, as samples were processed in batch and needed to be identified prior to sequence analysis.

Reporting for specific materials, systems and methods

We require information from authors about some types of materials, experimental systems and methods used in many studies. Here, indicate whether each material, system or method listed is relevant to your study. If you are not sure if a list item applies to your research, read the appropriate section before selecting a response.

Materials & experimental systems

n/a	Involved in the study
☒	☐ Antibodies
☐	☒ Eukaryotic cell lines
☒	☐ Palaeontology
☒	☐ Animals and other organisms
☒	☐ Human research participants
☒	☐ Clinical data

Methods

n/a	Involved in the study
☒	☐ ChIP-seq
☐	☒ Flow cytometry
☒	☐ MRI-based neuroimaging

Eukaryotic cell lines

Policy information about cell lines

Cell line source(s)	293T ATCC CRL-3216
Authentication	All genomic sites tested in this study were PCR amplified and verified against known HEK293T genomic sequences before conducting gene editing experiments to authenticate the cell lines.
Mycoplasma contamination	Cell lines were tested negative for mycoplasma contamination.
Commonly misidentified lines (See ICLAC register)	None used in this study.

Flow Cytometry

Plots

Confirm that:

- ☒ The axis labels state the marker and fluorochrome used (e.g. CD4-FITC).
- ☒ The axis scales are clearly visible. Include numbers along axes only for bottom left plot of group (a 'group' is an analysis of identical markers).
- ☒ All plots are contour plots with outliers or pseudocolor plots.
- ☒ A numerical value for number of cells or percentage (with statistics) is provided.

Methodology

Sample preparation	Bacterial cells were grown to an OD of 0.2 overnight and were diluted to equivalent concentrations in PBS for analysis.
Instrument	BD FACSAria™ III: 4 laser, 12 color sorter.
Software	BDFACS Diva software was used to collect data. Open source FCSalyzer (https://sourceforge.net/projects/fcsalyzer/) was used to analyze Flow Cytometry data.

Cell population abundance	10,000 gated events for data analysis were collected based on default FSC/SSC parameters for E. Coli.
Gating strategy	10,000 gated events for data analysis were collected based on default FSC/SSC parameters for E. Coli. The GFP+ gate was determined by both a "No dCas9" negative control and a "dSpyCas9" positive control. The gating strategy is provided in Supplementary Figure 4.

☒ Tick this box to confirm that a figure exemplifying the gating strategy is provided in the Supplementary Information.
