# Supplementary material for: A Cas9 with PAM recognition for adenine dinucleotides
Source: Nat Commun. 2020 May 18;11:2474. doi: 10.1038/s41467-020-16117-8 (PMC7235249; doi:10.1038/s41467-020-16117-8)
Supplement: Supplementary file 1 — Supplementary Information [file 41467_2020_16117_MOESM1_ESM.pdf]

Reviewers' Comments:

Reviewer #1:
Remarks to the Author:
Jakimo et al. report the characterization of a Cas9 (SmacCas9) from Streptococcus macacae and show that it has an NAA PAM preference. In principle this makes it a valuable addition to the genome editing effector line-up, since other Cas9s that are in frequent use all include at least one G or C residue, precluding edits in purely A/T-rich regions. Cas12a orthologs and engineered variants exist with exclusively A/T-containing PAMs, as the authors note, somewhat blunting the value-added conferred by SmacCas9, though the authors present some limited evidence that SmacCas9 is more active than these Cas12a effectors (also discussed further below).

For the most part, the work presented is fine as far as it goes, and most of the important points (such as PAM specificity) are supportable based on the evidence shown. However, the analyses are very thin in comparison with other recent reports of new Cas9 editors. Many of the approaches taken fall short of the usual standards of the field circa 2019. For instance, the rigorous quantitative analyses of PAM preferences in vitro, editing efficiencies in cells, and mutational spectra that are enabled by NGS readouts are never adopted, though they are now routine. T7E1 analyses alone can be misleading in reporting true editing efficiencies; among other ramifications, this renders the SmacCas9 vs. Cas12a activity comparisons less than definitive. Most of the editing events claimed from the data in Fig. S5B are unconvincing based on the barely visible gel bands from T7E1 digestion. Tests of editing efficacy are limited to plasmid transfections in transformed cells, and this sets a very low bar relative to the primary cell and in vivo contexts, and additional delivery modes, that the field needs to move genome editing applications forward. These issues significantly weaken the case for publication. This is unfortunate because this Cas9 may well indeed be useful to the field, once its activities, characteristics, and efficacies in more demanding contexts are rigorously and convincingly defined.

Reviewer #2:
Remarks to the Author:
In the manuscript entitled “A Cas9 with Preferred PAM Recognition for Adenine Dinucleotides”, Noah Jakimo et. al described a homolog of SpyCas9 in Streptococcus macacae with native 5’-NAA-3’ PAM specificity. Furthermore, they replaced the PI domain of SpyCas9 with that of Smac Cas9, generating a chimeric Spy-mac Cas9. The authors showed that Spy-mac Cas9 hybrids can target all adenine dinucleotide PAM sequences and possess robust editing efficiency in HEK293T cells. Although similar strategies have been reported for engineering Cas9 variants with modified PAM specificity, the newly discovered Spy-mac Cas9 is interesting as a useful gene editing tool, which expands the Cas9 targeting range to genomic sites with 5’-NAA-3’ PAM. Unfortunately the results are under-sampled that makes the conclusions unconvincing, and several comparisons are not performed with appropriate controls.

Major comments:
1. The authors used T7EI assay and Sanger sequencing to analyze the gene editing efficiency of different Cas9. However, both methods have been shown to be less accurate and prone to report artifacts such as binding without cutting. For example, I can barely see the cleaved bands in the T7EI assay in Figure S5 and the results are not convincing. Deep sequencing analysis is recommended to accurately evaluate the gene editing efficiency for all important data presented in the manuscript.

2. As shown in Figure S4G, it seems that the neighboring sequences of NAA PAM may affect the gene editing efficiency of Spy-mac Cas9. For instance, the cleavage efficiency is low at sites with the TAGAA and TATAA PAMs compared to others. Interesting, Spy-mac Cas9 seems to be active at sites with non-“NAA” PAM, such as TATAT and TTATA. Similarly, Spy-mac Cas9 only generated

very low indel (~<2.5%) at genomic targets with CAACCCCA, AAACCAGC, and AAATTCCA detected by T7EI assay in Fig. 3B. These results are inconsistent with the results shown in Figure 2B. Maybe more genomic sties should be included to draw a concrete explanation and conclusion. For Figure 3B, the statement on Page 7, "iSpy-mac Cas9 consistently generated a larger indel percentage than either AsCas12a or LbCas12a" seems to be incorrect.

3. Many data sets are incomplete. For example, on Page 8, the author noticed "a significant gain" compared to the indel formation when they used double-strand breaking enzymes. However, the double-strand breaking enzyme control are not included in the Figure 3C and S6. Besides, deep sequencing analysis should be used to accurately evaluate the base editing efficiency instead of Sanger sequencing.

Minor comments:
1. The species name and the 11 aa sequences around the PAM-contacting arginines of 115 orthologs of Spy Cas9 from UniProt should be listed.
2. In Page 5, Supplementary Figure S3F should be Supplementary Figure S4F.
3. In Page 6, "5′- or 3-" should be "5′- or 3′-".

RE: Ms. No. NCOMMS-19-17179
"Review: A Cas9 with Preferred PAM Recognition for Adenine Dinucleotides"

Reviewer 1

Major Comments

1. "Jakimo et al. report the characterization of a Cas9 (SmacCas9) from Streptococcus macacae and show that it has an NAA PAM preference. In principle this makes it a valuable addition to the genome editing effector line-up, since other Cas9s that are in frequent use all include at least one G or C residue, precluding edits in purely A/T-rich regions. Cas12a orthologs and engineered variants exist with exclusively A/T-containing PAMs, as the authors note, somewhat blunting the value-added conferred by SmacCas9, though the authors present some limited evidence that SmacCas9 is more active than these Cas12a effectors (also discussed further below).

   For the most part, the work presented is fine as far as it goes, and most of the important points (such as PAM specificity) are supportable based on the evidence shown. However, the analyses are very thin in comparison with other recent reports of new Cas9 editors. Many of the approaches taken fall short of the usual standards of the field circa 2019. For instance, the rigorous quantitative analyses of PAM preferences in vitro, editing efficiencies in cells, and mutational spectra that are enabled by NGS readouts are never adopted, though they are now routine. T7E1 analyses alone can be misleading in reporting true editing efficiencies; among other ramifications, this renders the SmacCas9 vs. Cas12a activity comparisons less than definitive. Most of the editing events claimed from the data in Fig. S5B are unconvincing based on the barely visible gel bands from T7E1 digestion. Tests of editing efficacy are limited to plasmid transfections in transformed cells, and this sets a very low bar relative to the primary cell and in vivo contexts, and additional delivery modes, that the field needs to move genome editing applications forward. These issues significantly weaken the case for publication. This is unfortunate because this Cas9 may well indeed be useful to the field, once its activities, characteristics, and efficacies in more demanding contexts are rigorously and convincingly defined."

We thank the reviewer for pointing out the deficiencies in the initial version of our manuscript. We have taken significant extra steps to fully characterize the genome editing activity of our enzymes in human cells. First and foremost, we have replaced the T7E1 readout for nuclease activity with NGS, gathering data that clearly demonstrates the significant editing capabilities of iSpyMac. SpyMac edits on most 5'-NAA-3' PAM targets with efficiencies between 10-30%, but iSpyMac greatly improves on these efficiencies with modification efficiencies between 30-70%, even when editing sites that SpyMac cannot access (i.e. the tested 5'-AAA-3' site). We decided to use SpyCas9 as the editing control, rather than Cas12a, as other factors may create discrepancies in a Cas12a vs. Cas9 comparison. The major point of our manuscript is to provide altered PAM targeting capabilities for Type-IIA CRISPR endonucleases. Additionally, we provide off-target analysis to highlight the mismatch tolerance of our enzymes.

Reviewer 2

1. "The authors used T7EI assay and Sanger sequencing to analyze the gene editing efficiency of different Cas9. However, both methods have been shown to be less accurate and prone to report artifacts such as binding without cutting. For example, I can barely see the cleaved bands in the T7EI assay in Figure S5 and the results are not convincing. Deep sequencing analysis is recommended to accurately evaluate the gene editing efficiency for all important data presented in the manuscript."

As mentioned above for Reviewer 1, we have replaced the T7E1 readout for nuclease activity with NGS,

gathering data that clearly demonstrates the significant editing capabilities of iSpyMac.

2. "As shown in Figure S4G, it seems that the neighboring sequences of NAA PAM may affect the gene editing efficiency of Spy-mac Cas9. For instance, the cleavage efficiency is low at sites with the TAGAA and TATAA PAMs compared to others. Interesting, Spy-mac Cas9 seems to be active at sites with non-"NAA" PAM, such as TATAT and TTATA. Similarly, Spy-mac Cas9 only generated very low indel (~<2.5%) at genomic targets with CAACCCCA, AAACCAGC, and AAATTCCA detected by T7EI assay in Fig. 3B. These results are inconsistent with the results shown in Figure 2B. Maybe more genomic sties should be included to draw a concrete explanation and conclusion. For Figure 3B, the statement on Page 7, "iSpy-mac Cas9 consistently generated a larger indel percentage than either AsCas12a or LbCas12a" seems to be incorrect."

We thank the reviewer for this suggestion, and have now tested 12 targets with various PAM sequences in 4 separate genomic loci (sequence information is available in Table S1). In addition to our base editing analysis, we cover every iteration of individual bases at the upstream and downstream flanking bases of the 5'-NAAN-3' PAM. More specifically, SpyMac edits on most 5'-NAA-3' PAM targets with efficiencies between 10-30%, but iSpyMac greatly improves on these efficiencies with modification efficiencies between 30-70%, even editing sites that SpyMac cannot access (i.e. the 5'-AAA-3' site). To note, we decided to use SpyCas9 as the editing control, rather than Cas12a, for our editing analysis, as other factors may create discrepancies in a Cas12a vs. Cas9 comparison, and due to the fact that the contribution our manuscript is to provide altered PAM targeting capabilities for Type-IIA CRISPR endonucleases.

3. "Many data sets are incomplete. For example, on Page 8, the author noticed "a significant gain" compared to the indel formation when they used double-strand breaking enzymes. However, the double-strand breaking enzyme control are not included in the Figure 3C and S6. Besides, deep sequencing analysis should be used to accurately evaluate the base editing efficiency instead of Sanger sequencing."

We have now included a double-strand breaking enzyme control with SpyCas9 on 5'-NGG-3' target. We hope that this dataset conducted with NGS provides definitive evidence of the efficient editing capabilities of our enzymes.

Minor Comments

1. "The species name and the 11 aa sequences around the PAM-contacting arginines of 115 orthologs of Spy Cas9 from UniProt should be listed."

We thank the reviewer for the suggestion, and will include this annotated information in a supplementary table.

2. "In Page 5, Supplementary Figure S3F should be Supplementary Figure S4F."

We have corrected this labeling error in the updated manuscript.

3. In Page 6, "5'- or 3-" should be "5'- or 3'-".

As this section was rewritten to accommodate the NGS data analysis, this error is no longer present.

We are grateful to both referees for their insightful critiques that have helped us greatly improve the manuscript.

Reviewers' Comments:

Reviewer #2:
Remarks to the Author:
1. In the revised manuscript, the authors provided Indel% analyzed by NGS in Fig. 3. However, the results were still under-sampled (only 11 genomic sites were analyzed in Fig. 3A). And it is confusing that the PAM sequences only three nucleotides of the PAM sequences are indicated in the Fig. 3A and the corresponding text, instead of the NAAN format in the rest of manuscript. It is necessary to keep consistent. For the same reason, ‘SpCas9’ in the figure legend of Fig. 3A should be ‘SpyCas9’.
2. I recommend the authors to redo Fig. 3C by using NGS analysis to accurately evaluate the base editing efficiency.
3. In line 114, ‘Supplementary Figure S3F’ should be ‘Supplementary Figure S4F’. This typo had been pointed out but not been revised.
4. Supplementary Figure S5 is not mentioned in the text.

RE: Ms. No. NCOMMS-19-17179A
"Review: A Cas9 with PAM Recognition for Adenine Dinucleotides"

Reviewer 2

1. In the revised manuscript, the authors provided Indel% analyzed by NGS in Fig. 3. However, the results were still under-sampled (only 11 genomic sites were analyzed in Fig. 3A). And it is confusing that the PAM sequences only three nucleotides of the PAM sequences are indicated in the Fig. 3A and the corresponding text, instead of the NAAN format in the rest of manuscript. It is necessary to keep consistent. For the same reason, ‘SpCas9’ in the figure legend of Fig. 3A should be ‘SpyCas9’.

Due to the COVID-19 situation, access to our MiniSeq machine for NGS was suspended soon after receiving the reviews. However, we thankfully managed to send in samples for two additional sites (for all nucleases), as well as the base editing samples, for sequencing to a commercial service provider before the lockdown. This additional data further supports our conclusion that iSpyMac is an efficient editor on 5’-NAAN-3’ PAMs. Furthermore, we have reverted the PAM sequences to reflect the 5’-NAAN-3’ format, and have fixed the figure legend to denote “SpyCas9” as requested.

2. I recommend the authors to redo Fig. 3C by using NGS analysis to accurately evaluate the base editing efficiency.

We have conducted NGS analysis for Fig. 3C and report the base editing results in the manuscript and in the figure set.

3. In line 114, ‘Supplementary Figure S3F’ should be ‘Supplementary Figure S4F’. This typo had been pointed out but not been revised.

We apologize for not fixing the typo in the previous revision. It has been fixed now.

4. Supplementary Figure S5 is not mentioned in the text.

We add a sentence in the manuscript where we summarize the data in Supplementary Figure S5 and reference it in the text.
