# Supplementary material for: A Cas9 with PAM recognition for adenine dinucleotides
Source: Nat Commun. 2020 May 18;11:2474. doi: 10.1038/s41467-020-16117-8 (PMC7235249; doi:10.1038/s41467-020-16117-8)
Supplement: Supplementary file 2 — Peer Review File [file 41467_2020_16117_MOESM2_ESM.pdf]

Supplementary Information

A Cas9 with PAM Recognition for Adenine Dinucleotides

Pranam Chatterjee[1,2,*], Jooyoung Lee[3], Lisa Nip[1,2], Sabrina R.T. Koseki[1,2], Emma Tysinger[1,2], Erik J. Sontheimer[3], Joseph Jacobson[1,2], and Noah Jakimo[1,2]

[1]MIT Center for Bits and Atoms, Massachusetts Institute of Technology, Cambridge, MA, USA
[2]MIT Media Lab, Massachusetts Institute of Technology, Cambridge, MA, USA
[3]RNA Therapeutics Institute, University of Massachusetts Medical School, Worcester, MA, USA

*Corresponding author

Supplementary Figures

1. Adenine nucleotide occurrences in human genome.
2. Comparisons of candidate orthologs.
3. CRISPR cassettes of candidate orthologs.
4. Flow cytometry gating strategy for PAM-SCANR assay.
5. tracrRNA determination for SpyMac.
6. Comparison of SpyMac to Cas12a as A-rich targeting platforms.

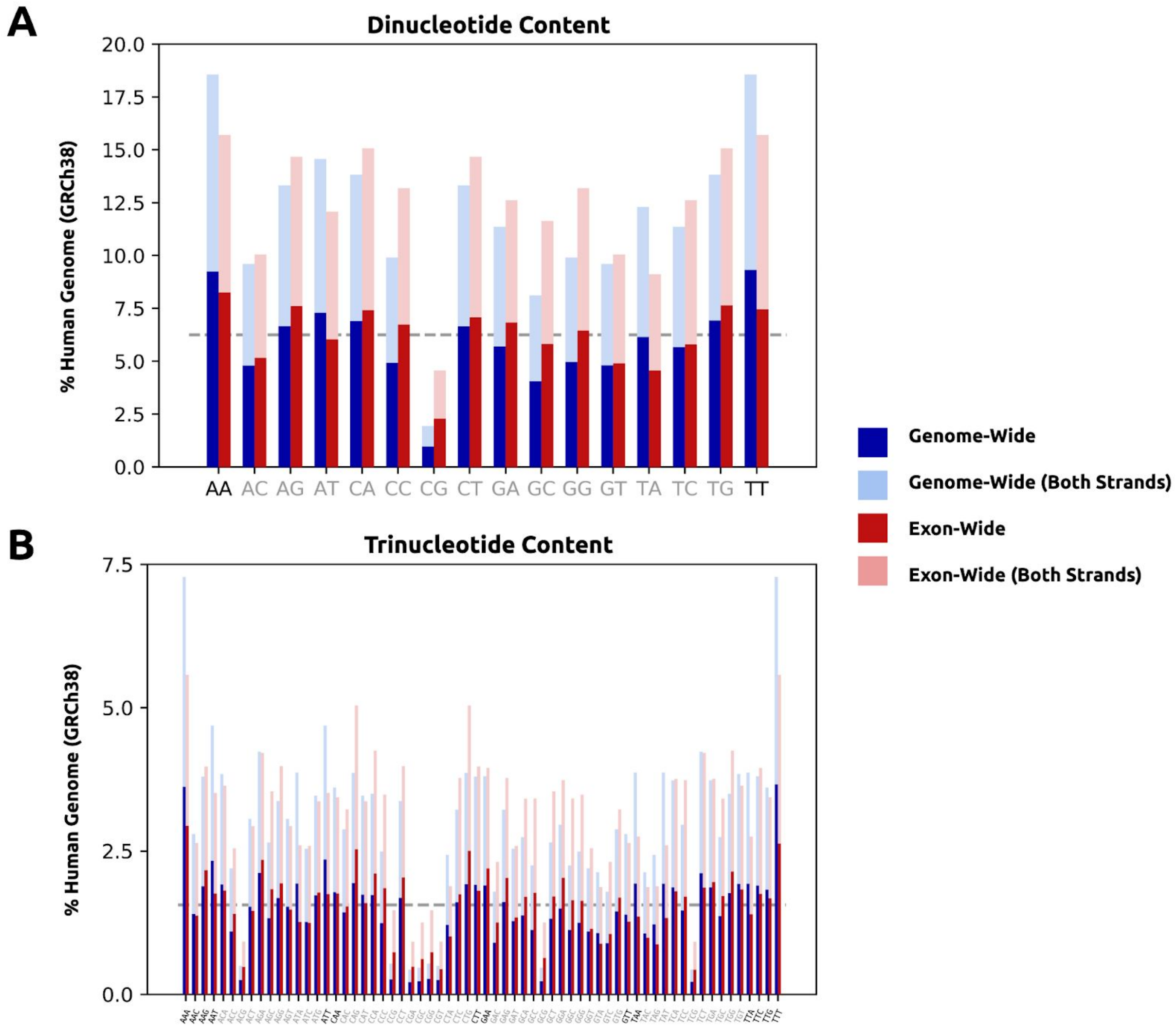

Supplementary Figure 1. (A) Dinucleotide and (B) Trinucleotide occurrences in the human reference genome GRCh38. Tallies were carried out using the compseq EMBOSS command line software tool. Dashed gray lines mark what the expected percentages would be for a uniform representation of all sequences of length 2 or 3.

A

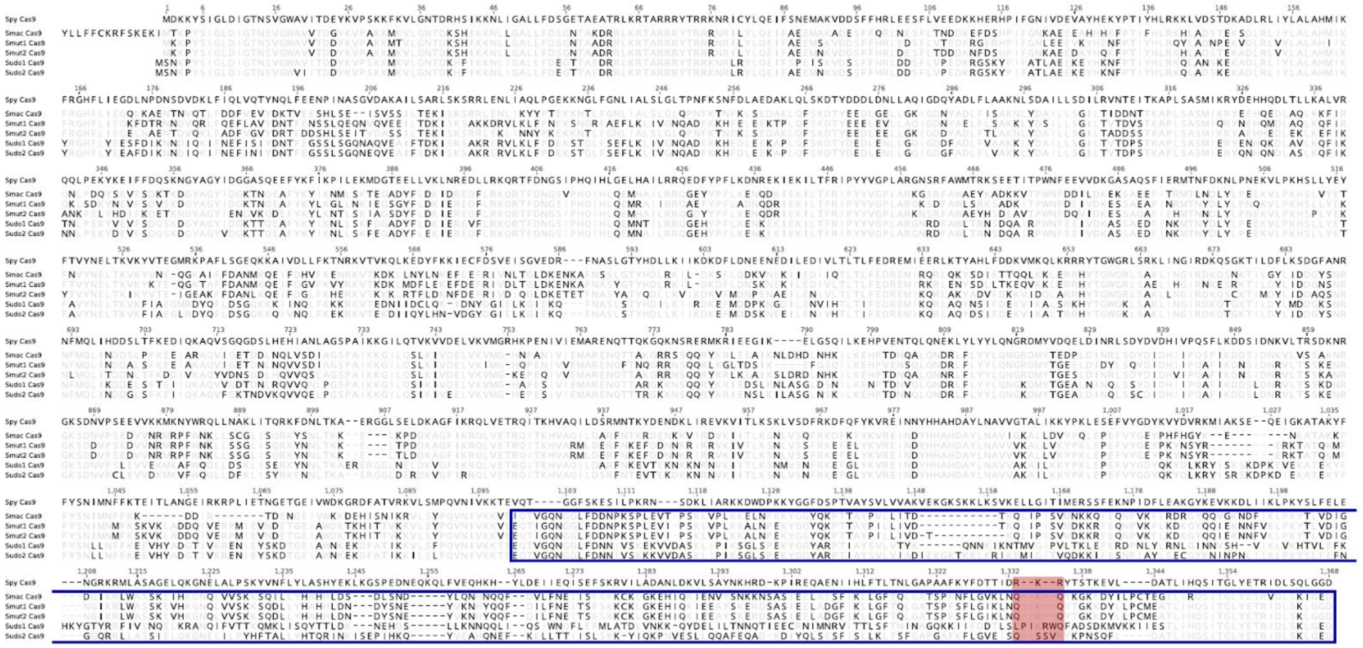

B

Ortholog of Interest	NCBI Accession	%Agreement to Spy1-1099	%Agreement to Spy1100-1368	%Agreement to Smac1100-1368
Smac Cas9	WP_003079701	64.6	37.9	-
Smut1 Cas9	BAQ19582	63.5	40.9	85.4
Smut2 Cas9	WP_024784288	65.7	40.9	85.4
Sudo1 Cas9	WP_049510439	64.4	30.2	39.9
Sudo2 Cas9	WP_049538452	64.8	48.8	46.8

S = *Streptococcus*, py = *pyogens*, mac = *macacae*, mut = *mutans*, udo = *pseudopneumoniae*

C

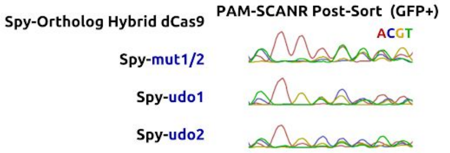

Supplementary Figure 2. (A) Sequence alignment (Geneious software) for selected orthologs of interest that substitute at least one critical PAM-contacting arginine residue within the region highlighted in red. A blue box marks the C-terminal component grafted onto truncated SpyCas9 to form dCas9 hybrids. (B) Table listing the homology shared within and outside of the box to those regions in the corresponding SpyCas9 and SmacCas9 reference sequences. (C) Chromatograms representing the PAM-SCANR based enrichment of variant-recognizing PAM sequences from a 5'-NNNNNNNN-3' library.

A

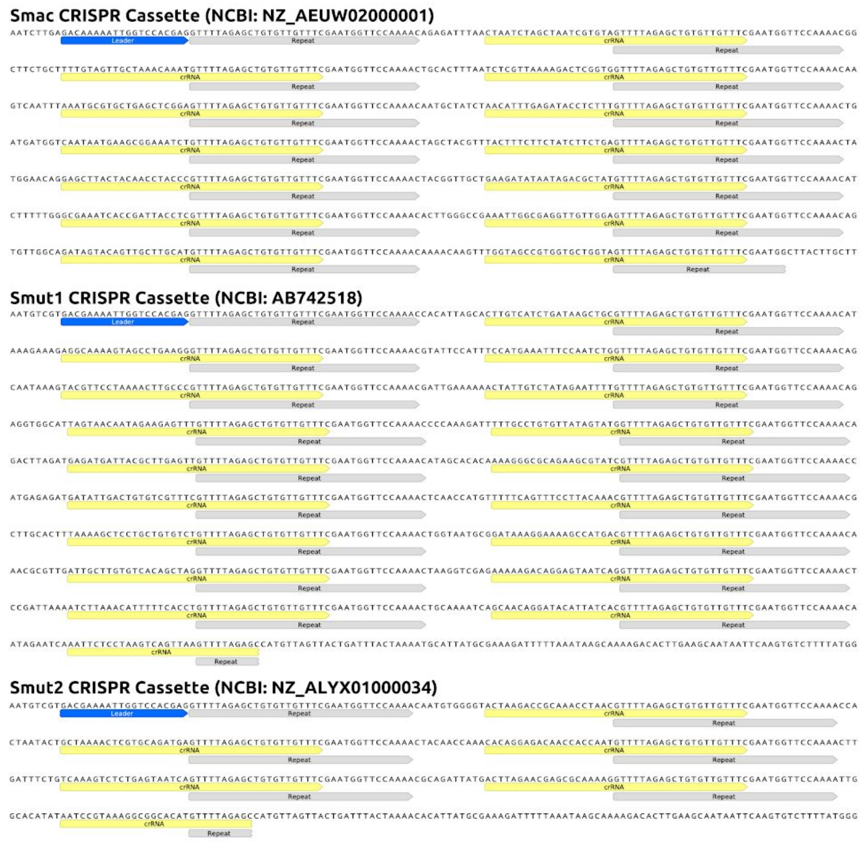

B

Notable Spacer Mappings

Putative Protospacer	PAM	Strain/Species Source
CTAATCTAGCTAATGGTGTA	TCAATTTTTT	*Macaca fascicularis* (Host; Primate)
AACATTTGAGATACCTCTTT	AAAGTAAGGC	*Macaca fascicularis* (Host; Primate)
GGGCAAAAGTAGCCTGAATT	CTAAGGATAG	*Macaca fascicularis* (Host; Primate)
TCCATGAAATTTCCAATCTG	TAAAGTCTGT	*Streptococcus phage APCM01/M102/M102AD*
TACGTTCCTAAAACTTGCCC	AAATCC----	*Parasteatoda tepidariorum* (House Spider)
GCGATGATTACGCTTGAGTT	AATAGAAATG	*Candidatus Izimaplasma* (Bacteria)
GATATTGACTGTGTCGTTTC	CAAATTCTCT	*Streptococcus phage M102AD*
TTTTCAGTTTCCTTACAAAC	TAAGATTTTC	*Streptococcus phage APCM01*
TTTTCGGTTTCCTTACAAAT	CAAGATTTTC	*Streptococcus phage M102/M102AD*
TAAAAGCTCCTGCTGTGTCT	TTAAGGTGCC	*Danio rerio* (Zebrafish)
GATAAAGGAAAAGCCATGAC	TCAACGCAAA	*Ovis canadensis* (Bighorn Sheep)
GATAAAGGCAAGGCCATGAC	CAAAGCCATC	*Streptococcus phage APCM01*
TGTGCTTGTGTCACAGCTAG	AAGGTTCTCT	*Lepisosteus oculatus* (Spotted Gar)
AAAAAGACAGGAGTAATCAG	TAAAAGATAC	*Pieris rapae* (Cabbage White Butterfly)
AAAAAAACAGGTGTAATCAG	CAAAACACTA	*Streptococcus phage M102*
ACTAAGACCGCCAACTTAAC	TAACTTGGAA	*Streptococcus phage M102/M102AD*
ACCAAGACCGCAAACTTAAC	TAACTTAGAA	*Streptococcus phage APCM01*
GCTAAAACCCGTGCAGATGA	TAACTTCGTT	*Streptococcus phage M102*
GCTAAAACTCGTGCAGATGA	TAATTTTGTT	*Streptococcus phage APCM01*
TCAAAGTTTCTGAGTAGTCA	TATATGTAAA	*Streptococcus phage M102/M102AD*
TCAGAGTCTCAGAATAGTCA	TATATGTAAA	*Streptococcus phage APCM01*

Supplementary Figure 3. (A) Annotated CRISPR cassettes obtained from the genomes corresponding to orthologs that substitute both PAM-contacting arginine residues to glutamine. (B) Mappings of CRISPR cassette spacers to their putative target source for listed crRNA, identified via an online BLAST and/or SPAMALOT. SPAMALOT uncovered most cases of mismatch-tolerated mappings to *Streptococcus phage*. Underlined bases indicate mismatches that are tolerated for the mapping. Additional line spacing separates analysis for each CRISPR cassette.

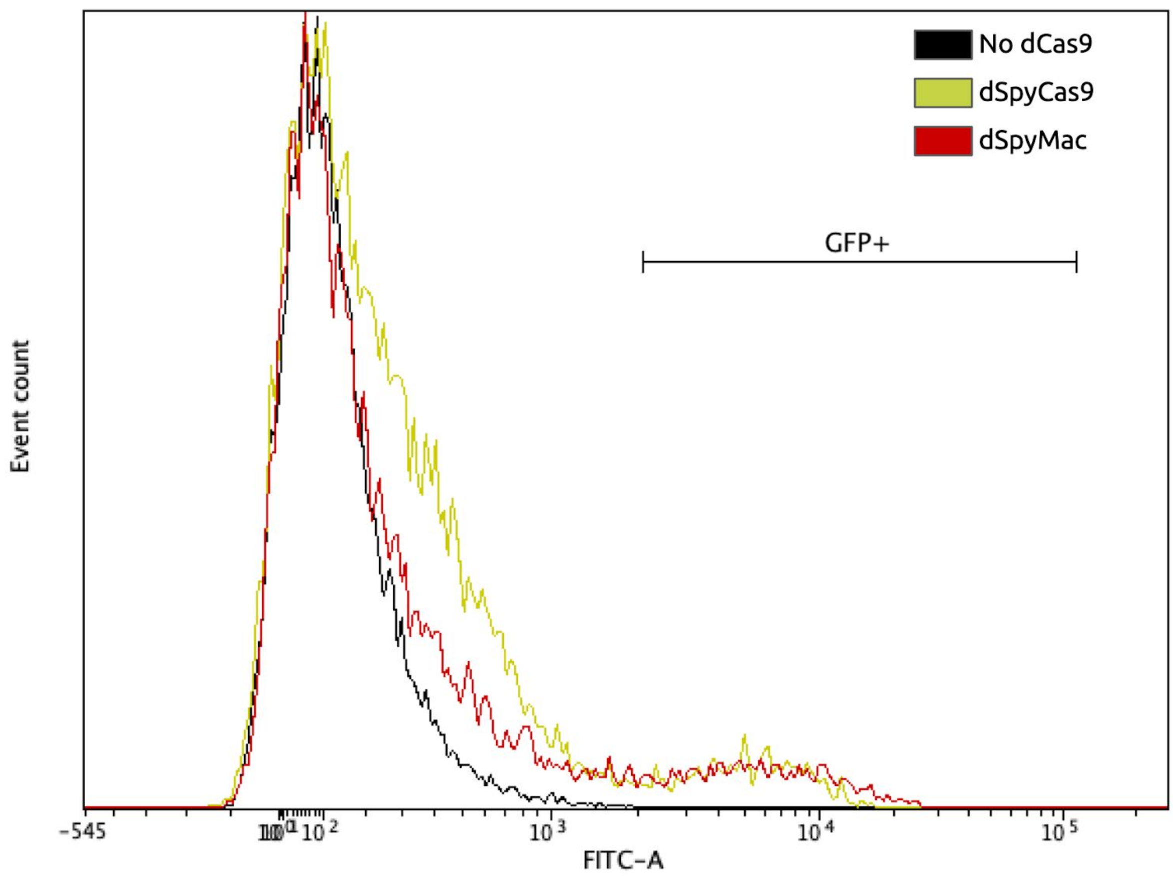

Supplementary Figure 4. 10,000 gated events for data analysis based on default FSC/SSC parameters for E. coli. The GFP+ gate was established both by a "no dCas9" negative control and a "dSpyCas9" positive control.

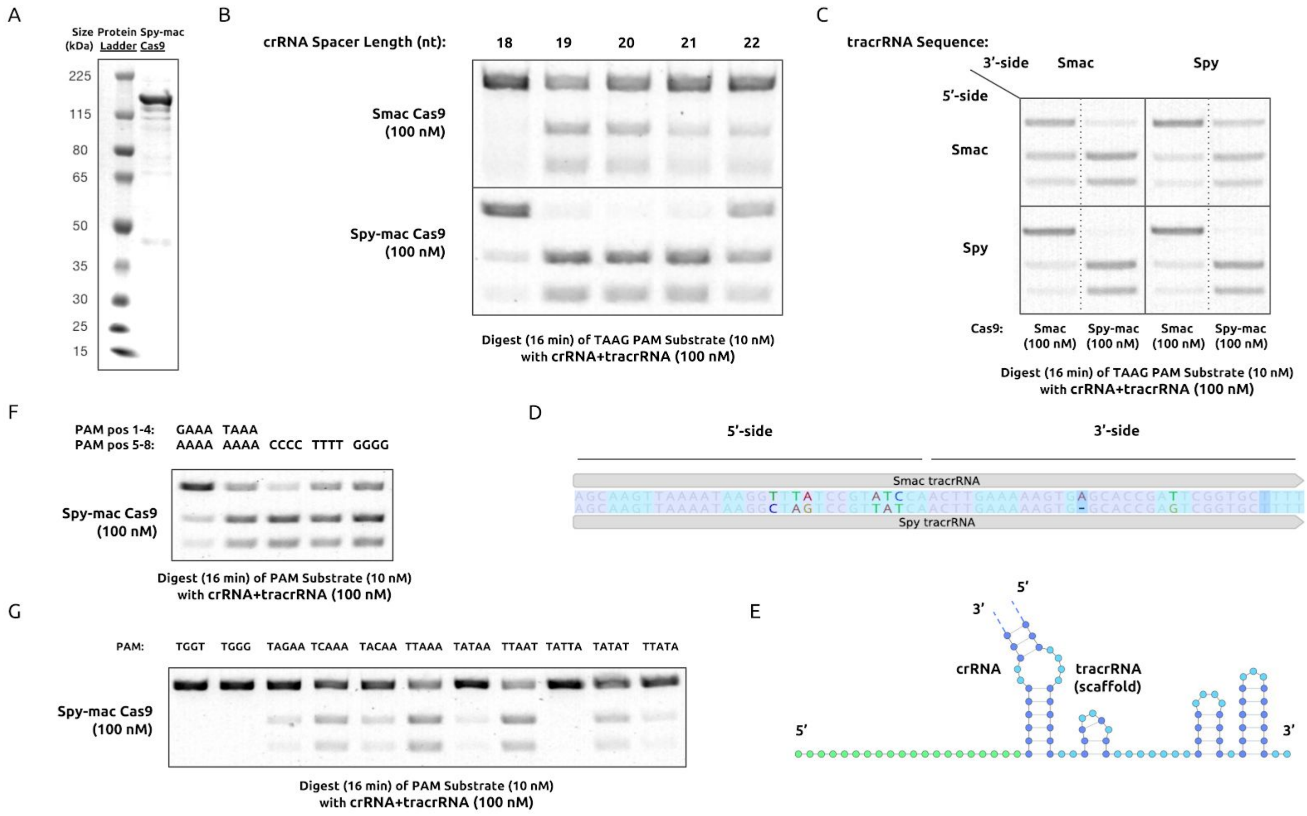

Supplementary Figure 5. (A) SDS-PAGE gel image of SpyMac after purification by affinity chromatography. SYBR-stained agarose gels running *in vitro* digestion reactions are shown that assay dependencies on (B) crRNA spacer length and (C) tracrRNA sequence origin. (D) Sequence alignment (Geneious software) of tracrRNA from *S. pyogenes* and *S. mutans* highlighted in a color code that reflects the base-pairing in their (E) duplex gRNA secondary structure. SYBR-stained agarose gels running *in vitro* digestion reactions are shown that assay dependencies on (F) positions 5-8 in the PAM sequence and (G) increments to the distribution of adenine content in positions 1-5 in the PAM sequence. All experiments in this figure were performed in independent biological duplicates (n=2), and experiments were repeated to ensure reproducibility.

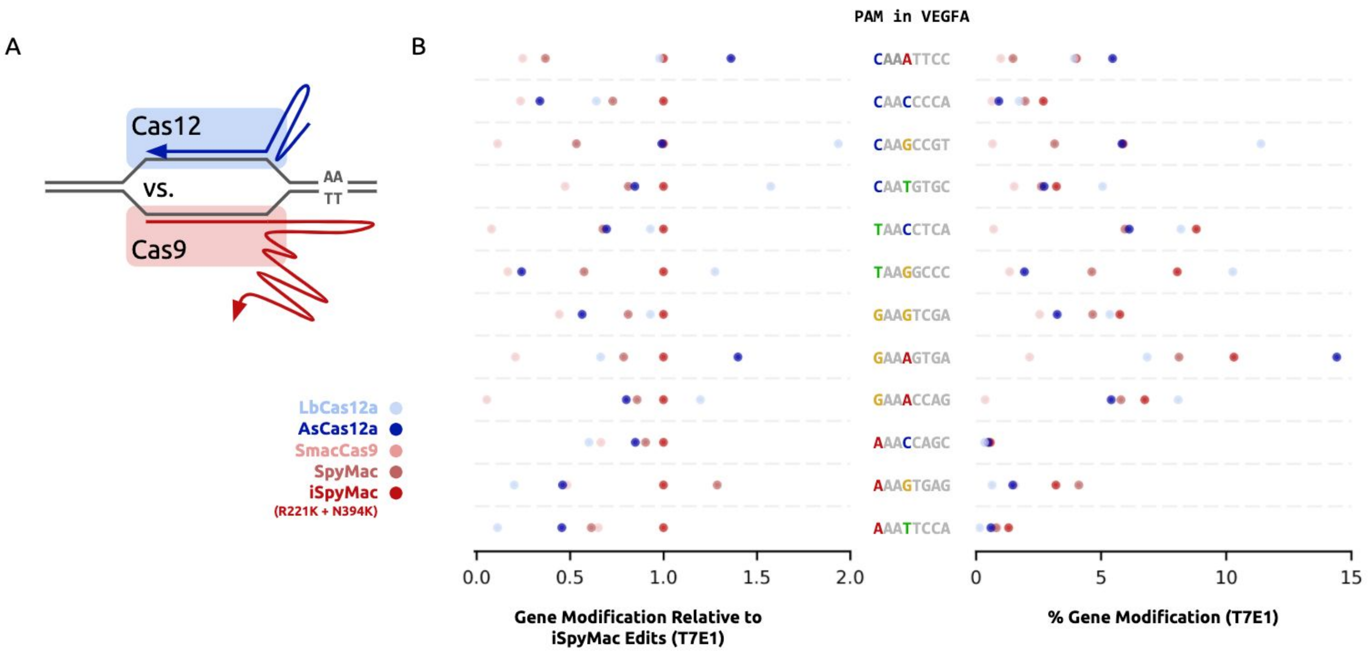

Supplementary Figure 6. (A) Schematic diagram for matching Cas9 and Cas12a guides in a manner that enforces their recognition of the same PAM sequence and therefore facilitates their comparison (a "Cas12a vs Cas9 Comparator"). (B) Dot plots of absolute and relative gene modification efficiency in HEK293T cells by Cas9 and Cas12a variants targeting common PAM sequences located in the *VEGFA* gene. Values were quantified in a T7E1-based assay and are consistent with two independent biological replicates (n=2) that were run in parallel. Source data are provided as a Source Data file.
